# Gene expression profiling in the synovium identifies a predictive signature of absence of response to adalimumab therapy in rheumatoid arthritis

**DOI:** 10.1186/ar2678

**Published:** 2009-04-23

**Authors:** Valérie Badot, Christine Galant, Adrien Nzeusseu Toukap, Ivan Theate, Anne-Lise Maudoux, Benoît J Van den Eynde, Patrick Durez, Frédéric A Houssiau, Bernard R Lauwerys

**Affiliations:** 1 Rheumatology Department, Cliniques Universitaires Saint-Luc, Université catholique de Louvain, Avenue Hippocrate 10, B-1200 Brussels, Belgium; 2 Rheumatology Department, CHU Brugmann, Place Arthur Van Gehuchten 4, 1020 Brussels, Belgium; 3 Pathology Department, Cliniques Universitaires Saint-Luc, Université catholique de Louvain, Avenue Hippocrate 10, B-1200 Brussels, Belgium; 4 Ludwig Institute for Cancer Research, Avenue Hippocrate 74, B-1200 Brussels, Belgium

## Abstract

**Introduction:**

To identify markers and mechanisms of resistance to adalimumab therapy, we studied global gene expression profiles in synovial tissue specimens obtained from severe rheumatoid arthritis (RA) patients before and after initiation of treatment.

**Methods:**

Paired synovial biopsies were obtained from the affected knee of 25 DMARD (disease-modifying antirheumatic drug)-resistant RA patients at baseline (T0) and 12 weeks (T12) after initiation of adalimumab therapy. DAS28-CRP (disease activity score using 28 joint counts-C-reactive protein) scores were computed at the same time points, and patients were categorized as good, moderate, or poor responders according to European League Against Rheumatism criteria. Global gene expression profiles were performed in a subset of patients by means of GeneChip Human Genome U133 Plus 2.0 Arrays, and confirmatory immunohistochemistry experiments were performed on the entire cohort.

**Results:**

Gene expression studies performed at baseline identified 439 genes associated with poor response to therapy. The majority (n = 411) of these genes were upregulated in poor responders and clustered into two specific pathways: cell division and regulation of immune responses (in particular, cytokines, chemokines, and their receptors). Immunohistochemistry experiments confirmed that high baseline synovial expression of interleukin-7 receptor α chain (IL-7R), chemokine (C-X-C motif) ligand 11 (CXCL11), IL-18, IL-18 receptor accessory (IL-18rap), and MKI67 is associated with poor response to adalimumab therapy. *In vitro *experiments indicated that genes overexpressed in poor responders could be induced in fibroblast-like synoviocytes (FLS) cultures by the addition of tumor necrosis factor-alpha (TNF-α) alone, IL-1β alone, the combination of TNF-α and IL-17, and the combination of TNF-α and IL-1β.

**Conclusions:**

Gene expression studies of the RA synovium may be useful in the identification of early markers of response to TNF blockade. Genes significantly overexpressed at baseline in poor responders are induced by several cytokines in FLSs, thereby suggesting a role for these cytokines in the resistance to TNF blockade in RA.

## Introduction

Tumor necrosis factor (TNF) antagonists are used routinely in severe rheumatoid arthritis (RA) patients who failed conventional disease-modifying antirheumatic drug (DMARD) therapy. According to large clinical trials, the three available drugs (adalimumab, infliximab, and etanercept) display similar effects in terms of efficacy, tolerability, and side effects [1-5]. These studies also indicate that about 25% of RA patients treated with TNF antagonists do not display any significant clinical improvement. Thus far, however, there are no validated tools that can predict whether an individual RA patient will respond to TNF blockade. Yet the identification of poor responders prior to initiation of therapy would direct the use of alternative methods of treatment, thereby preventing disease progression in these patients and saving unnecessary costs.

TNF antagonists interfere with many pathways involved in RA synovial inflammatory processes; these include local production of chemokines and cytokines [6-9], vascular proliferation and endothelial expression of adhesion molecules [10,11], cell trafficking into the synovium [8], proliferation of synovial macrophages [12-14], and production of matrix metalloproteinases [15]. Which of these pathways are critical in determining the clinical improvement associated with the use of TNF-blocking agents is still unknown. In the present study, we therefore wanted to investigate the effects of adalimumab on global gene expression changes in the RA synovium in order to obtain a molecular picture of the effects of TNF blockade in synovial tissue. We also investigated whether clinical, histological, and molecular characteristics of synovial biopsies at baseline are associated with response to therapy.

We harvested synovial biopsies in 25 severe RA patients followed prospectively before and 12 weeks after initiation of adalimumab therapy. Global gene expression studies and pathway analyses were performed in a subset of these patients, and confirmatory immunohistochemistry experiments were performed in the entire cohort. We found that adalimumab induces a significant decrease in the expression of genes involved in cell division in all patients. In responders, we also observed a decreased expression of genes involved in the regulation of immune responses (in particular, cytokines, chemokines, and their receptors). Moreover, we demonstrated that high baseline expression of selected genes from these families (cell division and regulation of immune responses) is associated with poor clinical response to therapy, thereby providing clinicians with potential tools to identify these patients prior to initiation of adalimumab treatment. Finally, we demonstrated that genes overexpressed in poor responders are induced in fibroblast-like synovial cell (FLS) cultures by the addition of several cytokines or combinations of cytokines: TNF-α, IL-1β, the association of TNF-α and IL-17, and the association of TNF-α and IL-1β.

## Materials and methods

### Patients and synovial biopsies

Twenty-five patients (18 women and 7 men, median age 55.2 years, range 18 to 83 years) with RA were included in the study. All patients met the American College of Rheumatology criteria for the diagnosis of RA [16]. Mean disease duration was 10 years (range 1 to 36 years). All patients had active disease at the time of tissue sampling and were resistant to conventional therapy. They all had erosive changes imaged on conventional x-rays of the hands and/or feet. All of them had a swollen knee at inclusion. Mean baseline serum C-reactive protein (CRP) level was 29.6 mg/L (range 5 to 122 mg/L), and mean baseline DAS28 (disease activity score using 28 joint counts)-CRP (three variables) evaluation was 5.55 (range 4.07 to 8.26). Twenty-two patients had positive anti-citrullinated cyclic peptide (anti-CCP2) antibody titers. All patients were treated with DMARDs, 23 with methotrexate (median dose 15 mg/week, range 7.5 to 20 mg/week), and 2 with leflunomide (20 mg/day); 18 of them were treated with low-dose steroids (prednisolone ≤ 7.5 mg/day). Six patients had been included in double-blind clinical trials before inclusion in the present study (1 in a Golimumab versus placebo trial, 3 in a MapKinase inhibitor versus placebo trial, and 2 in a TNF-α-converting enzyme [TACE] inhibitor versus placebo trial). These trials were stopped at least 3 months prior to initiation of TNF-blocking therapy. All drug dosages were stable from at least 3 months prior to initiation of TNF-blocking therapy until completion of the study. No steroid injections were allowed during the duration of the study.

Adalimumab therapy was initiated at a dosage of 40 mg subcutaneously every other week. Disease activity at baseline (T0) and 12 weeks after initiation of therapy (T12) was evaluated using DAS(28)-CRP (three and four variables) scores, and response to therapy was assessed according to the European League Against Rheumatism (EULAR) response criteria [17] that categorize patients as responders (good or moderate) and non-responders (or poor responders) based on changes in DAS activity between T0 and T12 and absolute DAS values at T12.

Synovial biopsies were obtained by needle arthroscopy of the affected knee of all patients at T0 and T12. For each procedure, four to eight synovial samples were snap-frozen in liquid nitrogen and stored at -80°C for later RNA extraction. The same amount of tissue was kept at -80°C for immunostaining experiments on frozen sections. The remaining material was fixed in 10% formalin and paraffin-embedded for conventional optical evaluation and immunostaining of selected markers. All of the experiments (RNA extraction, histology, and immunohistochemistry) were performed on at least four biopsies harvested during every procedure in order to correct for variations related to the potential heterogeneous distribution of synovial inflammation. The study was approved by the ethics committee of the Université catholique de Louvain, and informed consent was obtained from all patients.

### Fibroblast-like synoviocyte cultures

FLSs were purified from seven additional synovial biopsies from DMARD-resistant RA patients as previously described [18]. Briefly, minced synovial fragments were digested in 1 mg/mL hyaluronidase solution (Sigma-Aldrich, St. Louis, MO, USA) for 15 minutes at 37°C and 6 mg/mL collagenase type IV (Invitrogen, Paisley, UK) for 2 hours at 37°C. Next, cells were washed, resuspended in high-glucose Dulbecco's modified Eagle's medium (Invitrogen) supplemented with 1% antibiotics-antimycotics (Invitrogen) and 1% minimum essential medium sodium pyruvate (Invitrogen), and seeded at 10,000 cells per square centimeter in six-well plates. When the cells reached confluence, adherent cells were detached using sterile 0.5% trypsin-ethylenediaminetetraacetic acid (Invitrogen) and used as FLSs between passages 3 and 9. For the cytokine stimulation experiments, cells were seeded in 24-well plates at 25,000 per well. Unless stated otherwise, the following cytokine concentrations were used: TNF-α (R&D Systems, Minneapolis, MN, USA) 10 ng/mL, IL-1β (R&D Systems) 10 ng/mL, IL-6 (Peprotech, London, UK) 10 ng/mL, IL-7 (R&D Systems) 100 ng/mL, and IL-17 (R&D Systems) 50 ng/mL. After overnight incubation with the indicated cytokines, cells were harvested and total RNA was extracted using the Nucleospin^® ^RNA II extraction kit (Macherey-Nagel, Düren, Germany). RNA from some experiments was used for microarray hybridizations while the remaining material was used for cDNA synthesis and real-time polymerase chain reaction (PCR) experiments.

### Microarray hybridization

Total RNA was extracted from the synovial biopsies using the Nucleospin^® ^RNA II extraction kit (Macherey-Nagel), including DNase treatment of the samples. At least 1 μg of total RNA could be extracted from 12 samples at T0 and from 12 samples at T12 for further processing. Out of these 12 samples at T0 and 12 samples at T12, 8 originated from the same patients and were used in the paired analyses of gene expression before and after therapy. RNA quality was assessed using an Agilent 2100 Bioanalyzer and RNA nanochips (Agilent Technologies, Inc., Santa Clara, CA, USA). All samples had a 28s/18s ratio of greater than 1.8. Labeling of RNA (complementary RNA [cRNA] synthesis) was performed in accordance with a standard Affymetrix^® ^procedure (One-Cycle Target Labeling kit; Affymetrix UK Ltd., High Wycombe, UK); briefly, total RNA was first reverse-transcribed into single-stranded cDNA using a T7-Oligo(dT) Promoter Primer and Superscript II reverse transcriptase (RT). Next, RNase H was added together with *Escherichia coli *DNA polymerase I and *E. coli *DNA ligase, followed by a short incubation with T4 DNA polymerase in order to achieve synthesis of the second-strand cDNA. The purified double-stranded cDNA served as the template for the *in vitro *transcription reaction, which was carried out overnight in the presence of T7 RNA polymerase and a biotinylated nucleotide analog/ribonucleotide mix. At the end of this procedure, the biotinylated cRNA was cleaned and then was fragmented by a 35-minute incubation at 95°C.

GeneChip^® ^Human Genome U133 Plus 2.0 Arrays (spotted with 1,300,000 oligonucleotides informative for 47,000 transcripts originated from 39,000 genes) (Affymetrix UK Ltd.) were hybridized overnight at 45°C in monoplicates with 10 μg of cRNA. The slides then were washed and stained using the EukGE-WS2v5 Fluidics protocol on the GeneChip^® ^Fluidics Station (Affymetrix UK Ltd.) before being scanned on a GeneChip^® ^Scanner 3000. For the initial normalization and analysis steps, data were retrieved on Affymetrix GeneChip Operating Software (GCOS). The frequency of positive genes (genes with a flag present) was between 45% and 55% on each slide. After scaling of all probe sets to a value of 100, the amplification scale was reported to be inferior to 3.0 for all slides. The signals yielded by the poly-A RNA, hybridization, and housekeeping controls (glyceraldehyde-3-phosphate dehydrogenase [GAPDH] 3'/5' ratio of less than 2) were indicative of the good quality of the amplification and hybridization procedures.

The same protocol was used for the amplification and the hybridization of RNA obtained from cultured FLSs. One microgram of total RNA was used in the initial reaction. After the initial normalization steps on GCOS, the frequency of positive genes was between 42% and 45% on each slide. The amplification scale was inferior to 1.5 for all slides, and the GAPDH 3'/5' ratio was inferior to 1.3. The data discussed in this publication have been deposited in the Gene Expression Omnibus (GEO) of the National Center for Biotechnology Information [19] and are accessible through GEO series accession numbers [GEO:GSE15602] and [GEO:GSE15615].

### Quantitative real-time reverse transcriptase-polymerase chain reaction experiments

Quantitative real-time RT-PCR evaluation of lymphotoxin beta (*LTB*) [GenBank: NM_002341.1], chemokine ligand 5 (*CCL5*) [GenBank: NM_002985], and cytotoxic T-lymphocyte-associated antigen 4 (*CTLA4*) [GenBank: NM_005214.3] gene expression was performed in synovial biopsies at T0 and T12. cDNA was synthesized from a subset of RNA that originated from 10 samples at T0 and 8 samples at T12 using RevertAid Moloney murine leukemia virus RT (Fermentas, St. Leon-Rot, Germany) and Oligo(dT) primers. Quantitative RT-PCR was performed on a MyiQ single-color RT-PCR detection system (Bio-Rad Laboratories, Nazareth Eke, Belgium) using SYBR Green detection mix. For each sample, 5 ng of cDNA was loaded in triplicate with 1× SYBR Green Mix (Applied Biosystems, Foster City, CA, USA) and the following 10 mM primers: β-*actin*: 5'-ggcatcgtgatggactccg-3' and 3'-ctggaaggtggacagcga-5'; *LTB*: 5'-gaggaggagccagaaacagat-3' and 3'-tagccgacgagacagtagagg-5'; *CCL5*: 5'-catattcctcggacaccacac-3' and 3'-gatgtactcccgaacccattt-5'; and *CTLA4*: 5'-ctcttcatccctgtcttctgc-3' and 3'-gacttcagtcacctggctgtc-3'. The melting curves obtained after each PCR amplification confirmed the specificity of the SYBR Green assays. Relative expression of the target genes in the studied samples was obtained using the difference in the comparative threshold (ΔΔCt) method. Briefly, for each sample, we determined a value for the cycle threshold (Ct), which was defined as the mean cycle at which the fluorescence curve reached an arbitrary threshold. The ΔCt for each sample was then calculated according to the formula Ct_target gene _- Ct_actin_; ΔΔCt values then were obtained by subtracting the ΔCt of a reference sample from the ΔCt of the studied samples. Finally, the levels of expression of the target genes in the studied samples as compared with the reference sample were calculated as 2^-ΔΔCt^.

Quantitative evaluation of *IL-7R *[GenBank: NM_002185], *IL-6 *[GenBank: NM_00600], *INDO *[GenBank: NM_002164], *GTSE1 *[GenBank: NM_016426], *CDC2 *[GenBank: NM_001786.3], and *MKI67 *[GenBank: NM_002417.4] gene expression was similarly conducted in FLSs using the following primers: *IL-7R*: 5'-ttcttggaggatgcagctaaa-3' and 3'-aagcccaaccaacaaagagtt-5'; *IL-6*: 5'-gcccagctatgaactccttct-3' and 3'-tgaagaggtgagtggctgtct-5'; *INDO*: 5'-ggtcatggagatgtccgtaa-3' and 3'-accaatagagagaccaggaagaa-5'; *GTSE1*: 5'-acgtgaacatggatgacccta-3' and 3'-gttcgggaaccggattattta-3'; *CDC2*: 5'-ggtcaagtggtagccatgaaa-3' and 3'-ccaggagggatagaatccaag-5'; and *MKI67*: 5'-ccccaaccaaaagaaagtctc-3' and 3'-gactaggagctggagggctta-5'.

### Histopathology and immunohistochemistry on paraffin-embedded sections

Fresh synovial biopsy tissue samples (n = 25 at T0 and n = 25 at T12) were fixed overnight in 10% formalin buffer at pH 7.0 and embedded in paraffin for histological and immunohistochemical analyses. Serial histological sections were stained with hematoxylin and eosin and analyzed by two observers (CG and IT) who were blinded to the clinical data. The following parameters were evaluated: vascular hyperplasia, perivascular lymphoplasmocytic cell infiltrates, diffuse lymphoplasmocytic cell infiltrates, follicular structures, thickness of the synovial lining layer, macrophages, polymorphonuclear cell infiltrates, fibrinoid necrosis, and fibrosis. A global semi-quantitative score including the whole biopsy areas was given for these parameters (0 to 3 scale: 0 indicates absence and 3 indicates high level). A specific score was assigned for the hyperplasia of the synovial lining layer: 0 (indicates one or two cell layers), 1 (three or four), 2 (five or six), and 3 (at least seven). Inter-observer correlation (Spearman *r*) was greater than 85% for every parameter tested except for synovial hyperplasia, which scored at 75%.

Immunolabeling experiments were performed using a standard protocol. After removal of paraffin and inactivation of endogenous peroxidases with 0.3% H_2_O_2 _for 30 minutes at room temperature, sections were incubated in 10 mM sodium citrate buffer (pH 5.8) and heated in a bain-marie at 98°C for 75 minutes to retrieve the antigenic sites. Non-specific binding was blocked by a 30-minute incubation with 50 mM Tris-HCl (pH 7.4) containing 10% (vol/vol) normal goat serum and 1% (wt/vol) bovine serum albumin. Sections then were incubated overnight at 4°C with the primary antibody. The following antibodies were used: CD3 (Neomarkers, Westinghouse, CA, USA), CD20 (Biocare Medical, Concord, CA, USA), CD68 (DakoCytomation, Glastrup, Denmark), CD15 (Biocare Medica), MKI67 (DakoCytomation), IL-18 (MBL, Nagoya, Japan), and gp130 (Santa Cruz Biotechnology, Inc., Santa Cruz, CA, USA). After three washes in 50 mM Tris-HCl (pH 7.4), specifically bound antibodies were labeled for 1 hour at room temperature with Envision™ (DakoCytomation), and the activity of peroxidases was revealed by a 10-minute incubation with 0.5 mg/mL diaminobenzidine in Tris-HCl buffer. As a final step, sections were washed in tap water and lightly counterstained with hematoxylin.

### Immunohistochemistry on frozen sections

After initial blocking of endogenous peroxidases with a peroxidase-blocking reagent (DakoCytomation), frozen sections of the synovial biopsy samples were stained with primary antibodies for the following molecules: interleukin-7 receptor α chain (IL-7R) (Sigma-Aldrich), chemokine (C-X-C motif) ligand 11 (CXCL11) (also named *ITAC*, interferon-inducible T-cell alpha chemoattractant) (Abcam, Cambridge, UK), and IL-18 receptor accessory (IL-18rap) (Abnova, Taipei, Taïwan). After incubation with the primary antibody, slides were sequentially incubated with an EnVision horseradish peroxidase (HRP) rabbit or mouse secondary antibody conjugated to an HRP-labeled polymer (Dako EnVision+System; DakoCytomation) and diaminobenzidene-positive chromagen (DakoCytomation). The slides were subsequently counterstained with hematoxyin for further analyses.

### Quantitative scoring of immunostaining

Quantitative analysis of the immunostained sections was performed using ImageJ software [20] in accordance with the Digital Image Analysis process [21]. Six digitalized pictures (magnification × 400) were obtained for each slide by two operators (VB and A-LM) who were blinded to the identity of the specimens. Each picture included lining and sublining regions when possible. When the distribution of the staining was heterogeneous, the pictures were taken in order to be representative of the globality of the slide. The surface staining (S) and the surface of the nuclei (N) were determined for each image, and the area of staining then was normalized by calculating the ratio of surface staining to nuclei staining.

### Statistical analyses

Statistical analyses of the microarray data were first performed using TMEV 4.0 [22]. Differences in gene expression between T0 and T12 were evaluated using paired Student *t *tests after processing of the scaled data for elimination of the genes with a flag absent in more than half of the samples and selection of the 8,000 genes that displayed the widest inter-individual variations in the remaining genes. Further statistical analyses were performed using Genespring^® ^software (Agilent Technologies, Inc.). For each slide, scaled data were normalized to the 50th percentile value for each chip and to the median value for each gene. The data were assessed by analysis of variance (ANOVA) for identification of differential gene expression at T0 among good, moderate, and poor responders, with the minimal level of differential expression between good and moderate versus poor responders set at 1.5-fold. Data obtained from the FLS cultures were similarly analyzed on Genespring^®^, using the same normalization steps and statistical tests.

Pathway analyses were performed using GOstat [23], an application that finds statistically overrepresented Gene Ontology (GO) terms within a group of genes [24]. These analyses were restricted to the terms inside the 'biological process' group of gene ontologies. Additional pathway analyses were performed using DAVID (Database for Annotation, Visualization and Integrated Discovery) [25], an application that interrogates additional functional annotation databases (Kegg pathways, BioCarta, and InterPro) and finds overrepresented biological themes within a group of genes.

## Results

### Clinical responses

**Figure 1:**
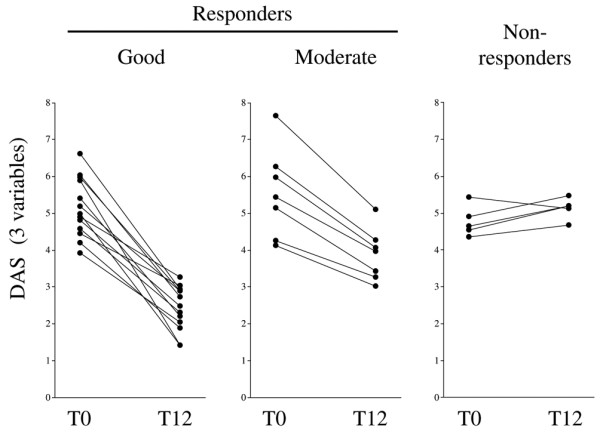
Evolution of disease activity score (DAS) (three variables) in 25 individual rheumatoid arthritis patients before (T0) and 12 weeks after (T12) initiation of adalimumab therapy. Patients are categorized into (good or moderate) responders or non-responders according to European League Against Rheumatism criteria.

Disease activity was prospectively evaluated at baseline (T0) and 12 weeks after initiation of adalimumab therapy (T12) based on DAS28-CRP (three variables) score evaluations. According to EULAR response criteria, 20 patients were responders at T12 (13 good and 7 moderate responders) whereas 5 were non-responders to adalimumab therapy (Figure 1). The use of DAS28-CRP (four variables) scores that include visual analog scale general health evaluation by the patient resulted in classification of the same 20 and 5 patients into responders versus non-responders, respectively. However, when this index was used among the responders, there were 11 good and 9 moderate responders.

We investigated whether baseline clinical characteristics were associated with response to therapy. DAS28-CRP (three variables) scores were not significantly different at baseline in responders (mean ± standard error of the mean [SEM]: 5.289 ± 0.213) and non-responders (mean ± SEM: 4.774 ± 0.186, *P *= 0.34). Similarly, DAS28-CRP (four variables) scores (mean ± SEM responders: 5.6725 ± 0.984; mean ± SEM non-responders: 5.066 ± 0.302, *P *= 0.19), CRP values (mean ± SEM responders: 27.9 ± 7.4 mg/L; mean ± SEM non-responders: 36.4 ± 21.4 mg/L, *P *= 0.64), and anti-CCP2 antibody titers (mean ± SEM responders: 477.2 ± 122.8 U/mL; mean ± SEM non-responders: 381.8 ± 208.7 U/mL, *P *= 0.72) were not significantly different in responders versus non-responders at baseline.

### Immunohistochemistry studies

First, we evaluated the effects of adalimumab therapy on the histopathological characteristics of the synovial biopsies harvested at T0 in a clinically affected knee and at T12. Semi-quantitative evaluation and paired comparisons of the biopsies indicated that adalimumab induced a significant decrease in the number of infiltrating polymorphonuclear cells between T0 and T12. By restricting the analyses to the biopsies from the 20 patients who responded to therapy, we could find evidence of a significant decrease in polymorphonuclear cell infiltration, fibrinoid necrosis, and diffuse lymphoplasmocytic cell infiltrates (data not shown).

**Figure 2:**
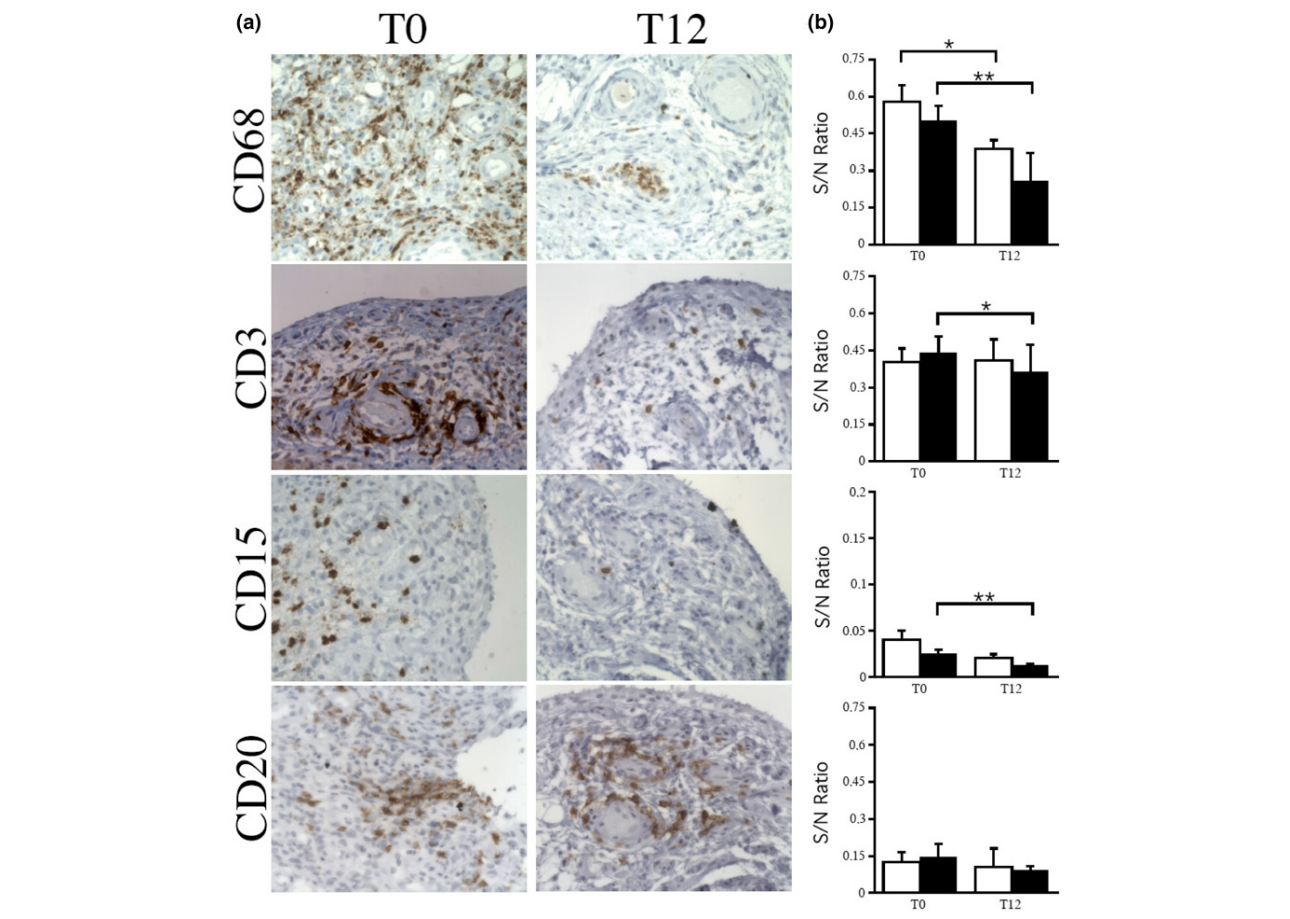
Changes in immunohistochemistry parameters in the synovial biopsies of severe rheumatoid arthritis patients. Biopsies were collected prior to (T0) (n = 25) and 12 weeks after (T12) (n = 25) initiation of adalimumab therapy. **(a) **Characteristic images of the stained markers (sublining C68, CD3, CD20, and CD15) (original magnification × 400). **(b) **Ratio of surface staining to staining of the nuclei (S/N). Slides stained for CD68, CD3, CD15, and CD20 were analyzed using ImageJ with six digitalized pictures (magnification × 400) obtained for each sample. Open boxes refer to all patients, and closed boxes refer to responders. Results are the mean and standard error of the mean of S/N ratio. *P < 0.05; **P < 0.005 versus good and moderate responders using Wilcoxon matched-pairs signed rank tests.

The effects of adalimumab on synovial cell populations were further investigated by immunohistochemistry. Quantitative analyses of CD68^+^, CD15^+^, CD3^+^, and CD20^+ ^cells and paired analyses indicated that adalimumab induced a significant decrease in the numbers of CD68^+ ^synovial cells in the sublining between T0 and T12 in all patients. When we considered the changes occurring only in the patients who responded to therapy, we found that adalimumab induced a significant decrease in the numbers of sublining CD68^+^, CD15^+^, and CD3^+ ^cells. By contrast, there were no changes in the numbers of CD20^+ ^cells (Figure 2).

We also investigated whether synovial immunohistochemistry parameters were different among the patients at T0, classified according to their EULAR response. ANOVAs comparing poor to moderate and good responders demonstrated that the amounts of fibrosis and fibrinoid necrosis were significantly higher in the synovial biopsies from the non-responders at baseline (data not shown). By contrast, we did not evidence any significant variation at T0 in the numbers of CD68^+^, CD3^+^, CD15^+^, and CD20^+ ^cells (evaluated by digital quantification) according to response to therapy.

### Effects of adalimumab therapy on synovial gene expression profiles

**Figure 3:**
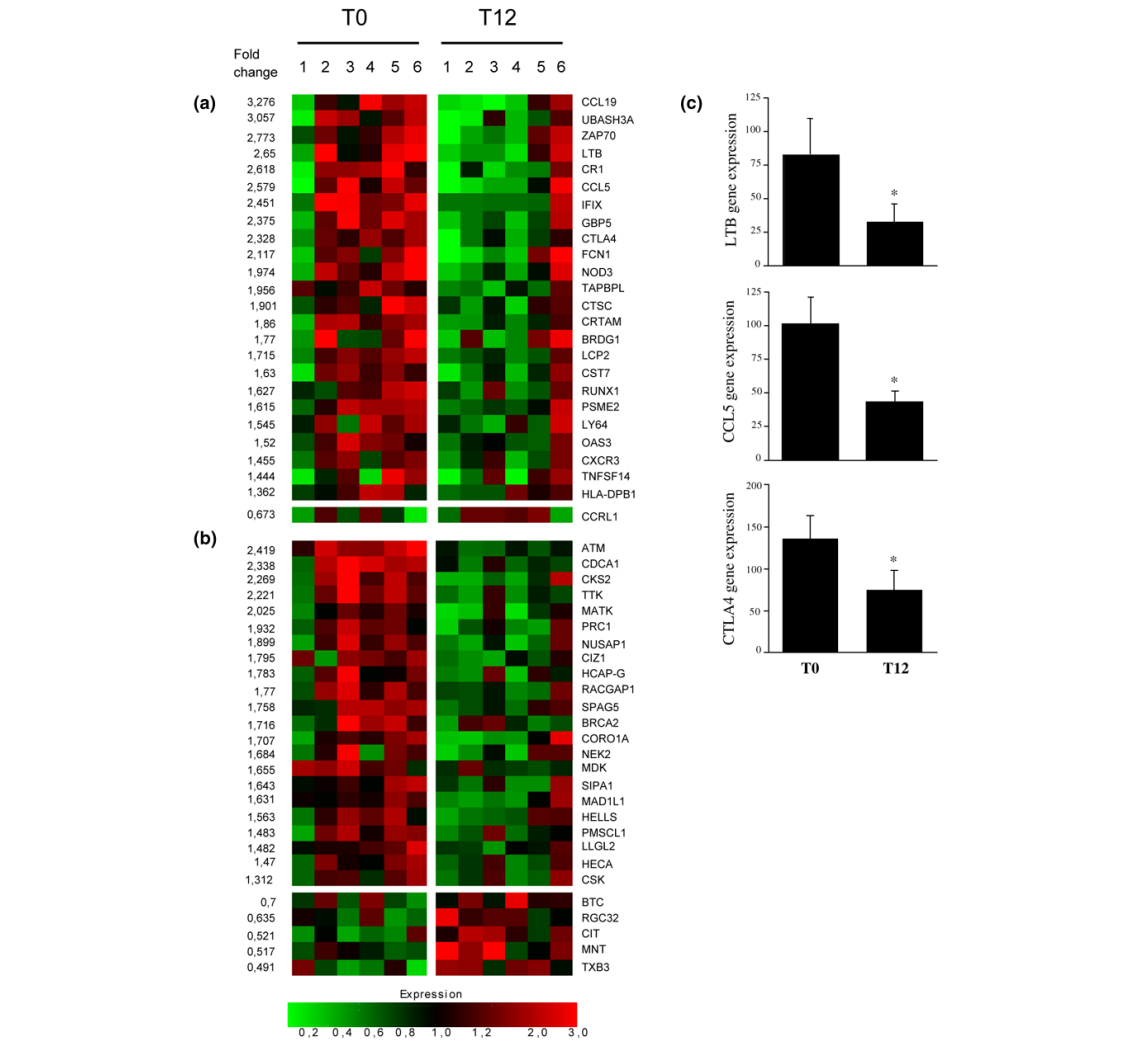
Genes differentially expressed before (T0) and 12 weeks after (T12) start of adalimumab in synovial biopsy specimens of rheumatoid arthritis patients who responded to therapy. Paired Student *t *tests indicated that 632 (out of 54,675) genes displayed significant differences in expression between T0 and T12 in six synovial tissue samples obtained from RA patients who responded to adalimumab therapy. Pathway analyses indicated that a significant percentage of these genes clustered into two distinct pathways: genes involved in the regulation of immune responses **(a) **and genes involved in cell division **(b)**. Fold-change values are the mean level of decreased expression at T12 as compared with T0. **(c) **Real-time reverse transcriptase-polymerase chain reaction studies of the expression of selected genes in rheumatoid arthritis synovial biopsy tissue before (T0) (n = 10) and 12 weeks after (T12) (n = 8) initiation of adalimumab therapy. Samples were loaded in triplicate, and results are the mean and standard error of the mean of gene expression, relative to the mean gene expression in a standard sample normalized to 1. *P < 0.05. CCL5, chemokine ligand 5; CTLA4, cytotoxic T-lymphocyte-associated antigen 4; LTB, lymphotoxin beta.

Next, we investigated the effects of adalimumab therapy on global gene expression profiles of synovial biopsies that were harvested at T0 and T12. RNA was extracted from eight synovial tissue samples at T0 and T12, labeled, and hybridized in monoplicates on GeneChip^® ^Human Genome U133 Plus 2.0 slides. According to paired Student *t *tests, 254 out of 54,675 transcripts were differentially expressed between T0 and T12 in all samples (Additional data file 1); 144 of them were downregulated and 110 were upregulated. To investigate whether these genes clustered in specific pathways, we analyzed the frequency of the available GO annotations in the list by means of online data-mining software. We found that genes differentially expressed between T0 and T12 were significantly enriched in GO families involved in cell division (9% of the GO annotated genes). If we restricted the analyses to the six patients who responded to therapy, we found 632 genes differentially expressed between T0 and T12. Interestingly, the latter genes clustered in two distinct families: genes involved in the regulation of immune responses and genes involved in the regulation of cell division (Figures 3a and 3b). To fine-tune these pathway analyses, we interrogated additional functional annotation databases (Kegg pathways, InterPro, and BioCarta) using DAVID. We found that the genes involved in the regulation of immune responses further distributed in pathways such as signal transduction, T-cell activation, antigen processing/presentation, and apoptosis. We confirmed our microarray data by performing real-time PCR evaluations of selected genes from the immune response gene families. As shown in Figure 3c, we found that *LTB*, *CCL5*, and *CTLA4 *gene expression was significantly lower at T12 as compared with T0.

### Correlation between clinical responses and gene signatures

**Figure 4:**
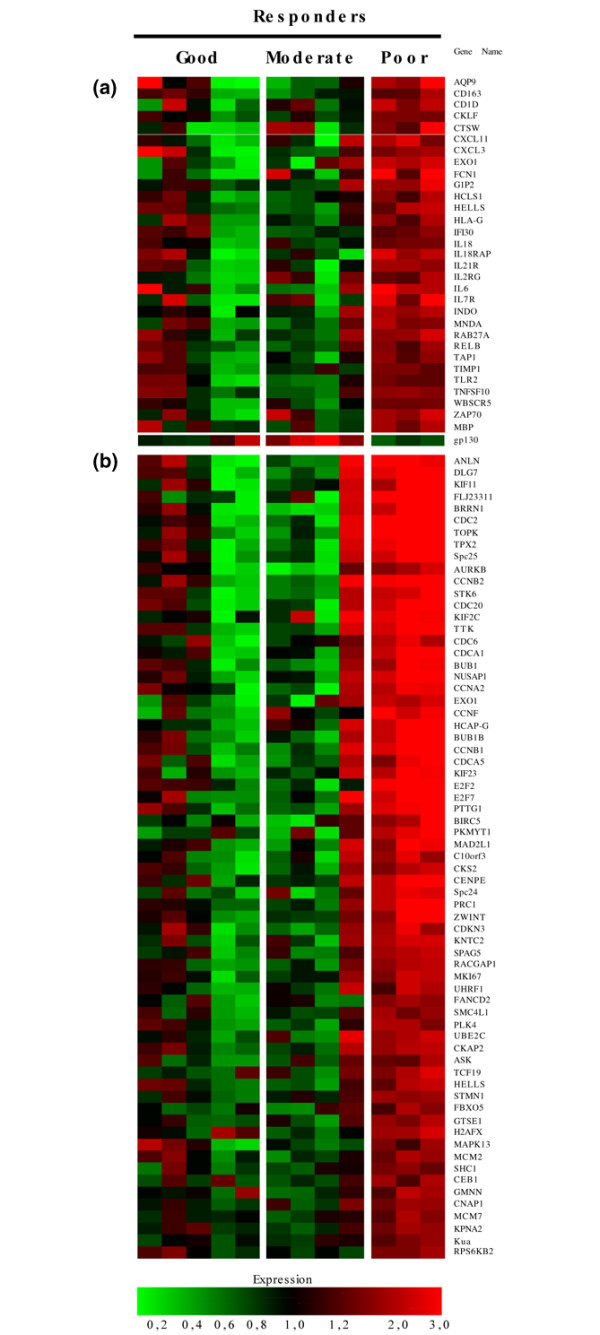
Genes differentially expressed at baseline between poor versus moderate and good responders to adalimumab therapy. Five hundred twenty-four genes were found to be differentially expressed among good, moderate, and poor responders at baseline by analysis of variance (*P *< 0.05). *Post hoc *(Student-Newman-Keuls) tests were used to discriminate genes that were specifically upregulated (n = 411) or downregulated (n = 28) in poor responders as compared with the two other groups. Pathway analyses indicated that these genes were significantly enriched in genes involved in the regulation of immune responses **(a) **and genes involved in cell division **(b)**.

We wondered whether clinical responses to therapy were associated with different patterns of gene expression at T0. We used ANOVA tests in order to identify genes differently expressed at T0 between 12 patients categorized as poor (3), moderate (4), and good (5) responders. We identified 524 genes that were differentially expressed between the three groups. In particular, 411 transcripts were found to be upregulated and 28 were downregulated in poor responders at T0 as compared with the two other groups. GO pathway analyses indicated that these genes were characterized by a distinct signature made of genes involved in the regulation of the cell cycle (28% of the GO annotated genes) and genes involved in the regulation of immune responses (15% of the GO annotated genes) (Figure 4). Interrogation of additional databases using DAVID indicated that the genes involved in the regulation of immune responses belong to pathways involved in the regulation of signal transduction, antigen processing/presentation, T-cell activation, and apoptosis.

**Figure 5:**
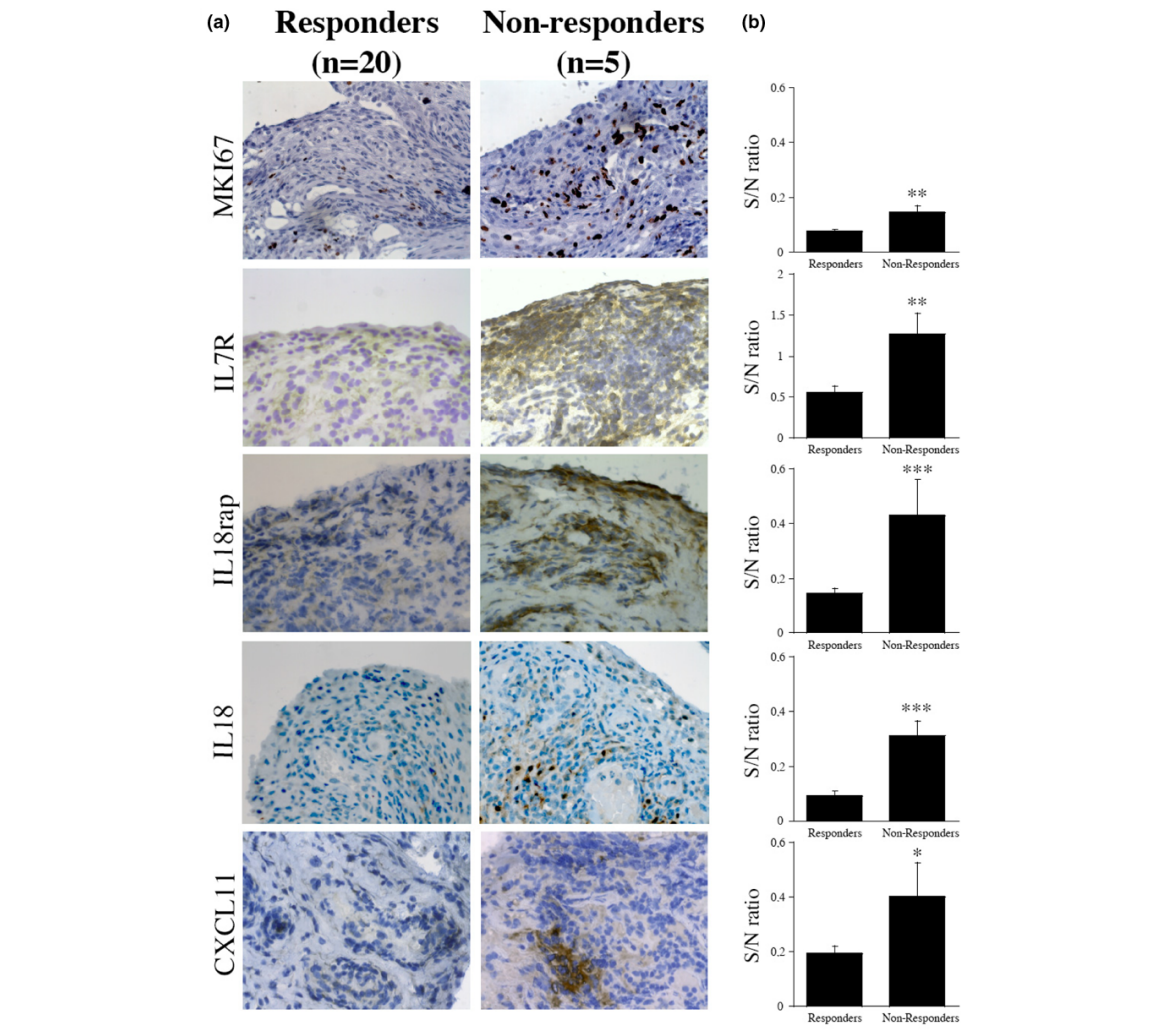
Baseline immunostaining for selected synovial markers of response to adalimumab therapy. Synovial samples of rheumatoid arthritis patients who responded or who did not respond to adalimumab therapy were stained at baseline with polyclonal antibodies directed at MKI67, interleukin-7 receptor α chain (IL-7R), interleukin-18 receptor accessory (IL-18rap), IL-18, and chemokine (C-X-C motif) ligand 11 (CXCL11). **(a) **Characteristic images of the stained markers are shown in responders (n = 20) versus non-responders (n = 5) (original magnification × 400). **(b) **Ratio of surface staining to staining of the nuclei (S/N). Slides were analyzed using ImageJ with six digitalized pictures (magnification × 400) obtained for each sample. Results are the mean and standard error of the mean of S/N ratio. *P < 0.05, **P < 0.005, ***P < 0.0005 using Wilcoxon matched-pairs signed rank tests.

To confirm our microarray findings related to differential gene expression at baseline depending on response to therapy, we performed immunostaining experiments on the synovial biopsy specimens obtained from the 25 patients included in the study. We evaluated the synovial expression of selected molecules from the immune response group at T0 using specific antibodies: IL-7R, CXCL11, IL-18, and IL-18rap. MKI67 was selected as a proliferation marker among the group of genes involved in the regulation of cell division. Quantitative evaluation of the slides confirmed that synovial expression of IL-7R, CXCL11, IL-18, IL-18rap, and MKI67 at T0 was significantly higher in poor as compared with moderate and good responders (Figure 5). There was no correlation between the digital quantifications of any of these molecules and cellularity markers (CD3, CD68, CD20, and CD15), thereby indicating that their synovial overexpression does not result from a shift in cell populations in non-responders.

### Genes overexpressed in poor responders are induced in fibroblast-like synoviocytes by the addition of several cytokines

**Figure 6:**
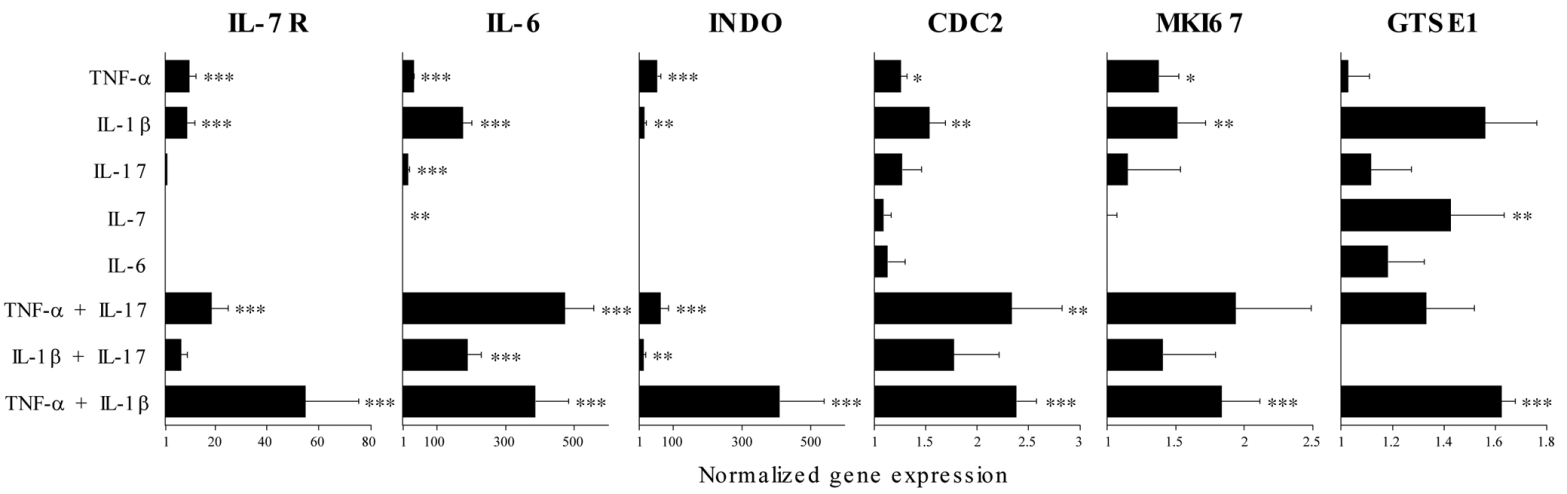
Genes overexpressed at baseline in poor responders are significantly induced by the combination of tumor necrosis factor-alpha (TNF-α) and interleukin-1β (IL-1β) in fibroblast-like synovial cells (FLSs). FLSs were cultured overnight in the presence of TNF-α (10 ng/mL), IL-1β (10 ng/mL), IL-6 (10 ng/mL), IL-7 (100 ng/mL), IL-17 (50 ng/mL), or combinations of several of these cytokines. RNA was extracted and real-time reverse transcriptase-polymerase chain reaction evaluation of *IL-7R*, *IL-6*, *INDO*, *CDC2*, *GTSE1*, and *MKI67 *was evaluated in at least four different experiments. Results are expressed as the mean fold change in gene expression and standard error of the mean, relative to the mean gene expression of the baseline condition normalized to 1. *P < 0.05, **P < 0.005, ***P < 0.0005 using Wilcoxon signed rank tests.

We wondered whether the genes overexpressed at T0 in non-responders were informative about synovial mechanisms of resistance to TNF blockade. In particular, we investigated whether these genes could be induced by TNF-α itself – which would indicate that their overexpression results from the overwhelming presence of TNF-α in the synovium – or whether they could be induced by other pro-inflammatory cytokines. FLSs were incubated overnight with TNF-α, IL-1β, IL-6, IL-7, IL-17, and combinations of these cytokines. Real-time PCR experiments were performed in order to study the expression of genes known to be overexpressed at baseline in poor responders (*IL-7R*, *IL-6*, *INDO*, *CDC2*, *GTSE1*, and *MKI67*). TNF-α alone, IL-1β alone, and the combination of TNF-α or IL-1β with IL-17 display stimulatory effects on some of the genes of this panel, whereas the combination of TNF-α and IL-1β had a significant stimulatory effect on the whole set of genes tested (Figure 6). Notably, the effects of the combination of TNF-α with either IL-17 or IL-1β were synergistic on several targets: IL-6 and CDC2 for TNF-α and IL-17, and IL-7R, IL-6, INDO, and CDC2 for TNF-α and IL-1β.

## Discussion

We studied synovial tissue from DMARD-resistant RA patients before and 12 weeks after initiation of therapy with adalimumab. Adalimumab therapy resulted in a significant decrease in the number of CD68^+ ^cells and in the expression of genes involved in cell division in all patients. In responders, we found a significant decrease in the numbers of CD68^+^, CD3^+^, and CD15^+ ^cells. From a gene expression point of view, responders were characterized by significant changes in the expression of genes involved in cell division and in the regulation of immune responses. Moreover, ANOVAs performed at baseline indicated that overexpression of selected genes belonging to both families was associated with poor response to therapy, an observation that was confirmed by immunostaining experiments. Finally, *in vitro *experiments performed in FLSs indicated that several cytokines and combinations of cytokines had a significant effect on the expression of a panel of genes overexpressed in poor responders at T0.

Several studies, aimed at the identification of prognostic markers of response to TNF blockade in RA, were recently published. Transcriptome analyses were performed recently by Sekiguchi and colleagues [26] in one study and by Lequerré and colleagues [27] in another study using peripheral blood mononuclear cells (PBMCs) from RA patients treated with infliximab. In a first set of 6 responders versus 7 non-responders, the latter identified 41 transcripts associated with response to therapy in baseline PBMC samples. They confirmed the association of 20 of these transcripts with response to therapy in an additional set of 20 patients [27]. It is striking, however, that the genes identified by these authors do not belong to any relevant pathway. It should be stressed in that perspective that RA is not a systemic disease. The inflammatory mechanisms targeted by TNF-blocking agents are located in the synovium, and gene expression profiles of RA PBMCs are not representative of these synovial tissue-specific pathways. In our previous studies, we found that transcriptomic analyses performed on synovial biopsies could discriminate RA from other joint disorders based on the analysis of synovial molecular profiles only, thereby demonstrating the power of this approach [28]. In this perspective, Lindberg and colleagues [29] investigated changes in global gene expression profiles in the synovium from a small group of RA patients before and after therapy with infliximab. They found a significant decrease in the expression of 1,058 genes in a subset of four patients with positive synovial immunostaining for TNF-α. These genes were enriched in families of genes involved in inflammatory processes.

Clinicians would be interested in measurable parameters that could predict response to TNF blockade prior to its initiation rather than in modifications of gene expression under therapy. Thus, van der Pouw Kraan and colleagues [30] performed global gene expression profiles in RA synovial tissue obtained in 6 non-responders and 12 responders prior to infliximab therapy. They found that responders were characterized by the overexpression of genes involved in specific pathways such as T-cell-mediated immunity, macrophage-mediated immunity, cytokine- and chemokine-mediated signaling pathways, major histocompatibility complex II-mediated immunity, and cell adhesion. Unfortunately, they did not perform any confirmatory experiment (real-time PCR or immunohistochemistry) in order to verify the reality of their microarray data [30]. Their results were also potentially biased by the fact that the synovial biopsies from the responders included in their study were characterized by higher percentages of CD3^+ ^and CD163^+ ^cells; therefore, it is not surprising that genes produced by these cells are overexpressed in tissues enriched for them. This kind of bias is very common in gene expression studies performed in heterogeneous tissues; in these studies, one must be aware that differences found in gene expression could be due to differences in cell populations across the samples rather than to true differences in pathogenic mechanisms at the single-cell level.

In the present study, we wanted to increase the validity of such microarray observations by performing additional RT-PCR and immunohistochemistry experiments and by linking our data to potential mechanisms of resistance to TNF blockade in RA. Our findings about the changes induced by adalimumab in synovial tissue between T0 and T12 are well in line with previous data from the literature. In particular, the significant decrease in CD68^+ ^cells is a well-documented characteristic of TNF-blocking agents in RA. Our gene expression studies show that adalimumab interferes with two major pathways of pathophysiological relevance in the RA synovium: regulation of immune responses and cell proliferation. Activation of these pathways is a major characteristic of the RA synovium [31,32], and our gene expression data confirm the well-documented role of TNF-α and TNF-blocking agents in the regulation of these pathogenic events.

The main interest of our study is that we identified significant differences in gene expression profiles at T0 according to the pattern of clinical response to therapy, while baseline clinical and histochemical characteristics were not different between responders and non-responders. In particular, we found that poor responders are characterized by a significant overexpression of genes involved in cell division and in the regulation of immune responses. The differential baseline expression of selected genes (*IL-7R*, *CXCL11*, *IL-18*, *IL-18rap*, and *MKI67*) among the samples (n = 25) was validated by immunostaining experiments, thereby qualifying them as potential predictive markers of response to adalimumab therapy in RA. The confirmation of these results in larger numbers of patients could result in the development of a diagnostic test to guide individualized therapy.

Strikingly, the genes overexpressed in poor responders are induced in FLSs by several cytokines, indicating that the absence of response could be due to the uncontrolled action of one or several of these cytokines. Earlier studies failed to demonstrate any correlation between synovial expression of TNF-α, IL-1β, or other cytokines and clinical response to TNF-blocking agents [33,34]. However, the biological effect of a cytokine results not only from the presence of the cytokine itself, but also from the concentration of its natural inhibitors (such as soluble TNF receptors or IL-1 receptor antagonists). Molecular signatures, therefore, are more suited to evaluate the biological action of a cytokine than raw evaluation of its synovial concentration.

By indicating that a representative panel of genes overexpressed in poor responders are induced in FLSs by TNF-α, IL-1β, and the combination of TNF-α with either IL-17 or IL-1β, our results raise the possibility that resistance to TNF blockade could be related to the effects of these cytokines on pro-inflammatory processes in poor responders. However, the study of gene expression signatures does not allow us to make strong mechanistic statements. Further experiments, therefore, are needed in order to test the *in vitro *sensitivity of synovial cells from TNF-blocking therapy-resistant patients to increasing concentrations of TNF-α-, IL-1β-, or IL-17-blocking agents and finally to identify the mechanisms of resistance to TNF blockade in RA.

## Conclusions

Using high-density oligonucleotide-spotted microarrays and immunohistochemistry experiments, we identified baseline markers of response to TNF blockade in a group of RA patients treated with adalimumab. We demonstrated that the genes overexpressed in the poor responders are induced by TNF-α, but also by IL-1β, in FLS cultures and by the combination of TNF-α with IL-17 or IL-1β, thereby suggesting that one (or several) of these cytokines plays a role in the mechanisms of resistance to adalimumab therapy. Our data also allow us to initiate larger studies in order to confirm the prognostic value of our markers in individual therapeutic decisions.

## Abbreviations

ANOVA: analysis of variance; anti-CCP2 antibody: anti-citrullinated cyclic peptide antibody (second-generation test); CCL5: chemokine ligand 5; cRNA: complementary RNA; CRP: C-reactive protein; Ct: cycle threshold; CTLA4: cytotoxic T-lymphocyte-associated antigen 4; CXCL11: chemokine (C-X-C motif) ligand 11; DAS: disease activity score; DAS28: disease activity score using 28 joint counts; DAVID: Database for Annotation, Visualization and Integrated Discovery; DMARD: disease-modifying antirheumatic drug; EULAR: European League Against Rheumatism; FLS: fibroblast-like synoviocyte; GAPDH: glyceraldehyde-3-phosphate dehydrogenase; GCOS: GeneChip Operating Software; GEO: Gene Expression Omnibus; GO: Gene Ontology; HRP: horseradish peroxidase; IL: interleukin; IL-18rap: interleukin-18 receptor accessory; IL-7R: interleukin-7 receptor α chain; LTB: lymphotoxin beta; PBMC: peripheral blood mononuclear cell; PCR: polymerase chain reaction; RA: rheumatoid arthritis; RT: reverse transcriptase; RT-PCR: reverse transcriptase-polymerase chain reaction; SEM: standard error of the mean; TNF: tumor necrosis factor.

## Competing interests

A patent application (WO 2008/132176) for the use of synovial markers as predictive markers of response to TNF blockade in RA was deposited by the Université catholique de Louvain (B.R. Lauwerys, B.J. Van den Eynde, Frédéric A. Houssiau and Valérie Badot). All other authors declare that they have no competing interests.

## Authors' contributions

VB helped to acquire, analyze, and interpret the data and helped to perform the statistical analyses and to write the manuscript. CG helped to acquire, analyze, and interpret the data. ANT, IT, A-LM helped to acquire the data. BJVdE, PD, and FAH helped to design the study and contributed to the writing of the manuscript. BRL helped to design the study and to acquire, analyze, and interpret the data and helped to perform the statistical analyses and to write the manuscript. All authors read and approved the final manuscript.

## Supplementary Material

Additional file 1 A table listing the genes differentially expressed between T0 and T12 in the synovium of adalimumab-treated RA patients. Microarray data were analyzed on TMEV 4.0 after elimination of the genes with a flag absent in more than half the samples and selection of the 8,000 genes that displayed the widest inter-individual variations. In all patients, 254 genes were found to display significant differences in expression between T0 and T12 using Student's t-tests. Fold changes are the ratio between mean expression at T0 above mean expression at T12. Click here for file
