# Supplementary material for: Gene expression profiling in the synovium identifies a predictive signature of absence of response to adalimumab therapy in rheumatoid arthritis
Source: Arthritis Res Ther. 2009 Apr 23;11(2):R57. doi: 10.1186/ar2678 (PMC2688209; doi:10.1186/ar2678)
Supplement: Additional file 1 — A table listing the genes differentially expressed between T0 and T12 in the synovium of adalimumab-treated RA patients. Microarray data were analyzed on TMEV 4.0 after elimination of the genes with a flag absent in more than half the samples and selection of the 8,000 genes that displayed the widest inter-individual variations. In all patients, 254 genes were found to display significant differences in expression between T0 and T12 using Student's t-tests. Fold changes are the ratio between mean expression at T0 above mean expression at T12. [file ar2678-S1.doc]

Supplementary data: Genes differentially expressed between T0 and T12 in the synovium of all adalimumab-treated RA patients.

| Genes down-regulated at T12 | |  |  |
| --- | --- | --- | --- |
|  |  |  |  |
| **Fold change** | **Name** | **GenBank** | **Description** |
| 5.01 | CENTB1 | NM_014716 | centaurin, beta 1 |
| 4.22 | NAP1L | AI763426 | napsin B pseudogene |
| 3.61 | MSC | AF060154 | musculin (activated B-cell factor-1) |
| 3.11 | LOC254531 | BG255923 | PlSC domain containing hypothetical protein |
| 3.00 | KIAA1442 | AL035460 |  |
| 2.81 | C11orf30 | NM_020193 | chromosome 11 open reading frame 30 |
| 2.62 | KIAA0540 | AB011112 | KIAA0540 protein |
| 2.58 | NFKB2 | NM_002502 | nuclear factor of kappa light polypeptide gene enhancer in B-cells 2 (p49/p100) |
| 2.53 | DEF6 | NM_022047 | differentially expressed in FDCP 6 homolog (mouse) |
| 2.51 | LTB | NM_002341 | lymphotoxin beta (TNF superfamily, member 3) |
| 2.48 | MRC2 | U58856 | mannose receptor, C type 2 |
| 2.38 |  | AI925316 | Transcribed sequence with weak similarity to protein ref:NP_060312.1 (H.sapiens) hypothetical protein FLJ20489 [Homo sapiens] |
| 2.37 | CDCA1 | AF326731 | cell division cycle associated 1 |
| 2.35 | IL21R | NM_021798 | interleukin 21 receptor |
| 2.35 | NAP1L | AI763426 | napsin B pseudogene |
| 2.32 |  | AC004692 | Homo sapiens PAC clone RP5-1107K12 from 7, complete sequence. |
| 2.31 | FLJ20174 | NM_017699 | hypothetical protein FLJ20174 |
| 2.29 | ADAM28 | NM_021777 | a disintegrin and metalloproteinase domain 28 |
| 2.29 | DCNP1 | AI141949 | dendritic cell nuclear protein 1 |
| 2.29 | BCL11A | AI912275 | B-cell CLL/lymphoma 11A (zinc finger protein) |
| 2.28 | HMMR | U29343 | hyaluronan-mediated motility receptor (RHAMM) |
| 2.23 | BIRC3 | U37546 | IAP homolog C; interacts with TRAF1 and TRAF2 in yeast two hybrid system; homolog of Baculovirus IAP genes; Mammalian IAP homolog C; Human IAP homolog C (MIHC) mRNA, complete cds. |
| 2.22 | MELK | NM_014791 | maternal embryonic leucine zipper kinase |
| 2.22 | MTAC2D1 | NM_152332 | membrane targeting (tandem) C2 domain containing 1 |
| 2.20 | PBX2 | NM_002586 | pre-B-cell leukemia transcription factor 2 |
| 2.20 | TTK | NM_003318 | TTK protein kinase |
| 2.19 |  | AL122039 | MRNA; cDNA DKFZp434E0572 (from clone DKFZp434E0572) |
| 2.17 | CTLA4 | AI733018 | cytotoxic T-lymphocyte-associated protein 4 |
| 2.16 | RPL18A | U52111 | Homo sapiens chromosome X clone Qc-7G6, QLL-F1720, QLL-C1335, Qc-8B7, Qc-11H12, Qc-7F6, QLL-E153, Qc-10E8, Qc-10B7 map q28, complete sequence. |
| 2.10 | JAK3 | BF512748 | Janus kinase 3 (a protein tyrosine kinase, leukocyte) |
| 2.09 |  | BC033052 | CDNA clone IMAGE:4770316, partial cds |
| 2.09 | SFRS14 | BG252853 | splicing factor, arginine/serine-rich 14 |
| 2.08 | GBP5 | BG271923 | guanylate binding protein 5 |
| 2.07 | EXOC7 | AK022397 | exocyst complex component 7 |
| 2.06 |  | AV764634 | AV764634 MDS Homo sapiens cDNA clone MDSBZE01 5', mRNA sequence. |
| 2.06 | FCN1 | NM_002003 | ficolin (collagen/fibrinogen domain containing) 1 |
| 2.06 | T3JAM | AL022398 |  |
| 2.06 | ITGAL | AC002310 |  |
| 2.02 | KLHL5 | AK001836 | kelch-like 5 (Drosophila) |
| 2.01 | ACSL4 | W95007 | acyl-CoA synthetase long-chain family member 4 |
| 1.99 | HLA-C | U62824 | major histocompatibility complex, class I, C |
| 1.99 | RCN3 | NM_020650 | reticulocalbin 3, EF-hand calcium binding domain |
| 1.98 | NUSAP1 | NM_016359 | nucleolar and spindle associated protein 1 |
| 1.98 | NUP210 | AA502912 | nucleoporin 210 |
| 1.95 | HCAP-G | NM_022346 | chromosome condensation protein G |
| 1.93 | FLJ14327 | NM_024912 |  |
| 1.92 | FNBP1 | BE046511 | formin binding protein 1 |
| 1.92 | PRC1 | NM_003981 | protein regulator of cytokinesis 1 |
| 1.92 | RASA4 | NM_006989 | DNA directed RNA polymerase II polypeptide J-related gene |
| 1.91 | MGC2647 | BF057084 | hypothetical zinc finger protein MGC2647 |
| 1.91 | BHC80 | AL832258 | BRAF35/HDAC2 complex (80 kDa) |
| 1.90 | ADAM28 | NM_021778 | a disintegrin and metalloproteinase domain 28 |
| 1.90 | FKBP10 | NM_021939 | FK506 binding protein 10, 65 kDa |
| 1.90 | C6orf37 | AL078599 | Human DNA sequence from clone RP5-991C6 on chromosome 6q14.1-15, complete sequence. |
| 1.90 | DOK1 | AF180527 | docking protein 1, 62kDa (downstream of tyrosine kinase 1) |
| 1.88 |  | M80469 | heavy chain; Human MHC class I HLA-J gene, exons 1-8 and complete cds. |
| 1.88 | NOD3 | AA694067 | NOD3 protein |
| 1.85 | EIF5A | BF541557 | CDNA FLJ46261 fis, clone TESTI4025062 |
| 1.85 | ORC6L | NM_014321 | origin recognition complex, subunit 6 homolog-like (yeast) |
| 1.84 | T3JAM | NM_025228 | TRAF3-interacting Jun N-terminal kinase (JNK)-activating modulator |
| 1.84 | PGF | BC001422 | placental growth factor, vascular endothelial growth factor-related protein |
| 1.83 | APAF1 | NM_013229 | apoptotic protease activating factor |
| 1.82 | LOC284021 | BG334495 | hypothetical protein LOC284021 |
| 1.81 |  | BF969544 | 602271878F1 NIH_MGC_84 Homo sapiens cDNA clone IMAGE:4360075 5', mRNA sequence. |
| 1.81 | LOC284009 | AL045793 | hypothetical protein MGC3329 |
| 1.81 | P4HB | NM_000918 | procollagen-proline, 2-oxoglutarate 4-dioxygenase (proline 4-hydroxylase), beta polypeptide (protein disulfide isomerase; thyroid hormone binding protein p55) |
| 1.80 | RASGEF1A | BF446578 | RasGEF domain family, member 1A |
| 1.80 | SPAG5 | NM_006461 | sperm associated antigen 5 |
| 1.80 | CENPA | NM_001809 | centromere protein A, 17kDa |
| 1.80 | RASSF4 | N49935 | Ras association (RalGDS/AF-6) domain family 4 |
| 1.79 | C19orf22 | AL574186 | hypothetical protein MGC16353 |
| 1.78 | DAPP1 | NM_014395 | dual adaptor of phosphotyrosine and 3-phosphoinositides |
| 1.78 | RPS14 | AF116710 | PRO2640 mRNA, complete cds |
| 1.78 | FKBP11 | NM_016594 | FK506 binding protein 11, 19 kDa |
| 1.77 | NELL2 | NM_006159 | NEL-like 2 (chicken) |
| 1.76 |  | T53962 | yb85d10.r1 Stratagene liver (#937224) Homo sapiens cDNA clone IMAGE:77971 5' similar to contains Alu repetitive element, mRNA sequence. |
| 1.76 | PRKCB1 | R51324 | yg72g01.s1 Soares infant brain 1NIB Homo sapiens cDNA clone IMAGE:38786 3', mRNA sequence. |
| 1.75 | MOV10 | BC002548 | Mov10, Moloney leukemia virus 10, homolog (mouse) |
| 1.75 | ETV7 | AF218365 | ets variant gene 7 (TEL2 oncogene) |
| 1.75 | FLJ25467 | BC036050 | KIAA1173 protein |
| 1.74 | RASA4 | AI738591 | polymerase (RNA) II (DNA directed) polypeptide J, 13.3kDa |
| 1.74 | BF | NM_001710 | B-factor, properdin |
| 1.74 | TACC3 | NM_006342 | transforming, acidic coiled-coil containing protein 3 |
| 1.74 | RACGAP1 | AU153848 | Rac GTPase activating protein 1 |
| 1.73 | C1GALT1 | AI672489 | wa03g11.x1 NCI_CGAP_Kid11 Homo sapiens cDNA clone IMAGE:2297060 3', mRNA sequence. |
| 1.72 | PPP1R16B | AB020630 | protein phosphatase 1, regulatory (inhibitor) subunit 16B |
| 1.72 | CKIP-1 | NM_016274 | CK2 interacting protein 1; HQ0024c protein |
| 1.72 | HLA-F | BE138825 | major histocompatibility complex, class I, F |
| 1.72 | C19orf6 | AI805266 | chromosome 19 open reading frame 6 |
| 1.72 | PASK | D50925 | PAS domain containing serine/threonine kinase |
| 1.71 | CDCA7 | AY029179 | cell division cycle associated 7 |
| 1.70 | BCL11A | BM193618 | B-cell CLL/lymphoma 11A (zinc finger protein) |
| 1.69 | PLEKHM1 | AJ002220 | pleckstrin homology domain containing, family M (with RUN domain) member 1 |
| 1.68 | KIAA0963 | AC005390 |  |
| 1.66 | HLA-G | M90684 | HLA-G histocompatibility antigen, class I, G |
| 1.65 | HIST2H2AA | NM_003516 | histone 2, H2aa |
| 1.62 | LAT | AF036906 | linker for activation of T cells |
| 1.62 | DOCK10 | AB014594 | dedicator of cytokinesis 10 |
| 1.62 | SCO2 | NM_005138 | SCO cytochrome oxidase deficient homolog 2 (yeast) |
| 1.62 | SIPA1 | NM_006747 | signal-induced proliferation-associated gene 1 |
| 1.61 | STAB1 | NM_015136 | stabilin 1 |
| 1.61 | BRIP1 | BF056791 | BRCA1 interacting protein C-terminal helicase 1 |
| 1.60 | PIGW | BF037819 | phosphatidylinositol glycan, class W |
| 1.59 | PTPNS1L3 | AK095499 | protein tyrosine phosphatase, non-receptor type substrate 1-like 3 |
| 1.59 |  | R12665 | CDNA FLJ27273 fis, clone TMS00761 |
| 1.57 | SPOCK2 | NM_014767 | synonym: testican-2; go_component: extracellular matrix [goid 0005578] [evidence NAS] [pmid 10386950]; go_function: calcium ion binding [goid 0005509] [evidence IDA] [pmid 10386950]; go_process: synaptogenesis [goid 0007416] [evidence NAS] [pmid 10386950]; go_process: extracellular matrix organization and biogenesis [goid 0030198] [evidence NAS] [pmid 10386950]; go_process: regulation of cell differentiation [goid 0045595] [evidence NAS] [pmid 10386950]; Homo sapiens sparc/osteonectin, cwcv and kazal-like domains proteoglycan (testican) 2 (SPOCK2), mRNA. |
| 1.57 | PSME2 | NM_002818 | proteasome (prosome, macropain) activator subunit 2 (PA28 beta) |
| 1.56 | LOC339903 | AA225165 | hypothetical protein LOC339903 |
| 1.55 | JAK2 | NM_004972 | Janus kinase 2 (a protein tyrosine kinase) |
| 1.55 | C20orf32 | BC027951 | chromosome 20 open reading frame 32 |
| 1.55 | INPP5D | NM_005541 | inositol polyphosphate-5-phosphatase, 145kDa |
| 1.54 | LY64 | NM_005582 | lymphocyte antigen 64 homolog, radioprotective 105kDa (mouse) |
| 1.54 | NALP1 | AF229062 | NACHT, leucine rich repeat and PYD containing 1 |
| 1.54 | LCP2 | AI123251 | lymphocyte cytosolic protein 2 (SH2 domain containing leukocyte protein of 76kDa) |
| 1.54 | DKFZp762C186 | AK092750 | tangerin |
| 1.53 | HIST1H2AC | AL353759 |  |
| 1.53 | BMP1 | NM_001199 | bone morphogenetic protein 1 |
| 1.53 |  | AL041761 | Clone IMAGE:4753714, mRNA |
| 1.52 | RASSF4 | AI890191 | Ras association (RalGDS/AF-6) domain family 4 |
| 1.51 | IL16 | NM_004513 | interleukin 16 (lymphocyte chemoattractant factor) |
| 1.51 |  | AL834519 | MRNA; cDNA DKFZp434N0220 (from clone DKFZp434N0220) |
| 1.50 | EZH2 | NM_004456 | enhancer of zeste homolog 2 (Drosophila) |
| 1.50 |  | BE350312 | Transcribed sequences |
| 1.50 | HELLS | AF155827 | helicase, lymphoid-specific |
| 1.49 |  | AU146329 | Transcribed sequences |
| 1.49 |  | AL512701 | CDNA FLJ39866 fis, clone SPLEN2015276 |
| 1.47 | CACNA2D4 | AI433691 | calcium channel, voltage-dependent, alpha 2/delta subunit 4 |
| 1.45 | SORL1 | AA290609 | sortilin-related receptor, L(DLR class) A repeats-containing |
| 1.45 | PMSCL1 | AI346350 | polymyositis/scleroderma autoantigen 1, 75kDa |
| 1.45 | LOC150271 | R54042 | LOC388888 (LOC388888), mRNA |
| 1.45 | LOC285533 | AW051591 | hypothetical protein LOC285533 |
| 1.44 | TNFRSF1B | NM_001066 | tumor necrosis factor receptor superfamily, member 1B |
| 1.44 | DGKQ | N45308 | yz17a12.s1 Soares_multiple_sclerosis_2NbHMSP Homo sapiens cDNA clone IMAGE:283294 3', mRNA sequence. |
| 1.44 | SDF2L1 | NM_022044 | stromal cell-derived factor 2-like 1 |
| 1.43 | LBR | NM_002296 | lamin B receptor |
| 1.42 | VAMP1 | NM_016830 | vesicle-associated membrane protein 1 (synaptobrevin 1) |
| 1.41 | DDA3 | BC001425 |  |
| 1.39 | KIAA1618 | AA976354 | oq48f08.s1 NCI_CGAP_Kid5 Homo sapiens cDNA clone IMAGE:1589607 3' similar to contains Alu repetitive element;, mRNA sequence. |
| 1.38 | HSPA6 | X51757 |  |
| 1.36 | MGC11102 | BC005131 | hypothetical protein MGC11102 |
| 1.35 | PSCD4 | AF125349 | pleckstrin homology, Sec7 and coiled-coil domains 4 |
| 1.35 | TUBA1 | AL565074 | tubulin, alpha 1 (testis specific) |
| 1.33 | CTSH | NM_004390 | cathepsin H |
| 1.25 | CYP1B1 | AU154504 | cytochrome P450, family 1, subfamily B, polypeptide 1 |
|  |  |  |  |
|  |  |  |  |
| **Genes up-regulated at T12** | | |  |
|  |  |  |  |
| **Fold change** | **Name** | **GenBank** | **Description** |
| 0.81 | FLJ12649 | NM_024597 |  |
| 0.72 | KIAA0367 | AB002365 | KIAA0367 |
| 0.71 | BC008967 | BE299456 | hypothetical gene BC008967 |
| 0.71 | DDR2 | AI799915 | discoidin domain receptor family, member 2 |
| 0.71 | PLSCR4 | NM_020353 | phospholipid scramblase 4 |
| 0.70 |  | AY010114 | Unknown mRNA sequence |
| 0.70 | C5orf4 | NM_016348 | chromosome 5 open reading frame 4 |
| 0.67 | C5orf13 | NM_004772 | chromosome 5 open reading frame 13 |
| 0.67 | DDR2 | W73819 | discoidin domain receptor family, member 2 |
| 0.66 | C6orf142 | AI242549 | qi17b04.x1 Soares_NhHMPu_S1 Homo sapiens cDNA clone IMAGE:1856719 3', mRNA sequence. |
| 0.66 | FLJ11078 | NM_018316 | hypothetical protein FLJ11078 |
| 0.66 | C14orf78 | AI935123 | chromosome 14 open reading frame 78 |
| 0.66 |  | AW973410 | Full length insert cDNA clone YU07D01 |
| 0.65 | GNG12 | N32508 | guanine nucleotide binding protein (G protein), gamma 12 |
| 0.65 |  | AK098337 | Homo sapiens cDNA FLJ41018 fis, clone UTERU2018881. |
| 0.65 | C2orf23 | BE535746 | hypothetical protein FLJ13110 |
| 0.65 | STEAP2 | BF680588 | six transmembrane epithelial antigen of prostate 2 |
| 0.64 | FLJ10847 | NM_018242 | hypothetical protein FLJ10847 |
| 0.64 | C20orf17 | AW953679 | chromosome 20 open reading frame 17 |
| 0.63 | ALDH1A3 | NM_000693 | aldehyde dehydrogenase 1 family, member A3 |
| 0.63 | LOC162073 | AI458417 | hypothetical protein LOC162073 |
| 0.61 |  | AA628481 | Transcribed sequences |
| 0.61 | MGC42630 | AI950007 | hypothetical protein MGC42630 |
| 0.60 | DNCI2 | AI823600 | dynein, cytoplasmic, intermediate polypeptide 2 |
| 0.60 | ESDN | AA805633 | endothelial and smooth muscle cell-derived neuropilin-like protein |
| 0.60 | FLNC | NM_001458 | filamin C, gamma (actin binding protein 280) |
| 0.59 | PRO1843 | NM_018507 |  |
| 0.59 | UBE2H | AA916831 | ubiquitin-conjugating enzyme E2H (UBC8 homolog, yeast) |
| 0.59 | EPM2AIP1 | BF432224 | EPM2A (laforin) interacting protein 1 |
| 0.59 | SGCD | AA479286 | sarcoglycan, delta (35kDa dystrophin-associated glycoprotein) |
| 0.58 | BTEB1 | AI690205 | basic transcription element binding protein 1 |
| 0.58 | ARHGAP24 | NM_031305 | hypothetical protein DKFZp564B1162 |
| 0.57 | G3BP | AA026297 | ze91g12.s1 Soares_fetal_heart_NbHH19W Homo sapiens cDNA clone IMAGE:366406 3' similar to contains Alu repetitive element;, mRNA sequence. |
| 0.57 |  | AK000176 | CDNA FLJ20169 fis, clone COL09536 |
| 0.56 | DCN | AI336924 | decorin |
| 0.56 | PCF11 | AI675753 | pre-mRNA cleavage complex II protein Pcf11 |
| 0.56 | PTPRD | N73931 | za74a07.s1 Soares_fetal_lung_NbHL19W Homo sapiens cDNA clone IMAGE:298260 3', mRNA sequence. |
| 0.56 | DKFZp434D1428 | N49941 | hypothetical protein DKFZp434D1428 |
| 0.55 | YWHAE | AA502643 | tyrosine 3-monooxygenase/tryptophan 5-monooxygenase activation protein, epsilon polypeptide |
| 0.55 | FLJ35801 | AI806805 | hypothetical protein FLJ35801 |
| 0.55 | AKAP12 | BF511276 | A kinase (PRKA) anchor protein (gravin) 12 |
| 0.55 | MGA | BF438227 | MAX gene associated |
| 0.55 | PRDM1 | AW977527 | PR domain containing 1, with ZNF domain |
| 0.55 | CSAD | BC018042 | cysteine sulfinic acid decarboxylase |
| 0.55 | HOXA10 | AI375919 | homeo box A10 |
| 0.54 | FLJ39963 | AK097282 | hypothetical protein FLJ39963 |
| 0.54 |  | AA322245 | Transcribed sequences |
| 0.53 |  | BE222109 | hu05h12.x1 NCI_CGAP_Lu24 Homo sapiens cDNA clone IMAGE:3165767 3', mRNA sequence. |
| 0.53 | SCAMP1 | NM_004866 | secretory carrier membrane protein 1 |
| 0.53 | ADAMTSL1 | NM_052866 | ADAMTS-like 1 |
| 0.53 | FAM38B | NM_022068 | hypothetical protein FLJ23403 |
| 0.52 | SULF1 | BE500977 | sulfatase 1 |
| 0.52 | PGK1 | AK055928 | phosphoglycerate kinase 1 |
| 0.52 |  | T86629 | CDNA clone IMAGE:5286019, partial cds |
| 0.51 | TAZ | H99038 | thyroid hormone receptor associated protein 3 |
| 0.51 | PDZRN4 | NM_013377 | similar to semaF cytoplasmic domain associated protein 3 |
| 0.51 |  | AI950302 | Transcribed sequences |
| 0.51 | ARHGEF7 | AI040887 | Rho guanine nucleotide exchange factor (GEF) 7 |
| 0.50 | COCH | BC007230 | coagulation factor C homolog, cochlin (Limulus polyphemus) |
| 0.50 | LOC115294 | BE150929 | RC4-HT0276-100300-015-e11 HT0276 Homo sapiens cDNA, mRNA sequence. |
| 0.50 | MGC16943 | AI688141 | similar to RIKEN cDNA 4933424N09 gene |
| 0.50 |  | BC036698 | CDNA clone IMAGE:4838650, partial cds |
| 0.50 | H326 | AA707411 | H326 |
| 0.50 | USP7 | BF433061 | ubiquitin specific protease 7 (herpes virus-associated) |
| 0.50 | ZNF145 | AW244016 | zinc finger protein 145 (Kruppel-like, expressed in promyelocytic leukemia) |
| 0.49 |  | AL049232 | Homo sapiens mRNA; cDNA DKFZp564P1816 (from clone DKFZp564P1816). |
| 0.49 | TK2 | AA524412 | thymidine kinase 2, mitochondrial |
| 0.49 | C1QTNF3 | NM_030945 | C1q and tumor necrosis factor related protein 3 |
| 0.49 | RREB1 | BF591556 | ras responsive element binding protein 1 |
| 0.48 | PPP3CA | AI827550 | protein phosphatase 3 (formerly 2B), catalytic subunit, alpha isoform (calcineurin A alpha) |
| 0.48 | USP25 | AF419247 | runt-related transcription factor 1 (acute myeloid leukemia 1; aml1 oncogene) |
| 0.48 | HRB | AI742626 | HIV-1 Rev binding protein |
| 0.48 | PDGFA | AW205919 | platelet-derived growth factor alpha polypeptide |
| 0.47 | TLE4 | AI742932 | transducin-like enhancer of split 4 (E(sp1) homolog, Drosophila) |
| 0.47 | LRRC16 | BE671038 | leucine rich repeat containing 16 |
| 0.47 | MGC34648 | AI631072 | hypothetical protein MGC34648 |
| 0.47 | MLLT4 | AI480107 | myeloid/lymphoid or mixed-lineage leukemia (trithorax homolog, Drosophila); translocated to, 4 |
| 0.46 |  | AK025909 | CDNA: FLJ22256 fis, clone HRC02860 |
| 0.46 | FLJ13611 | BI668018 | hypothetical protein FLJ13611 |
| 0.45 |  | AI344149 | Transcribed sequences |
| 0.45 |  | AV719355 | AV719355 GLC Homo sapiens cDNA clone GLCEMB06 5', mRNA sequence. |
| 0.44 | DAAM1 | AA890373 | dishevelled associated activator of morphogenesis 1 |
| 0.44 | XTP3TPB | AI821589 | XTP3-transactivated protein B |
| 0.44 | EMS1 | N45140 | ems1 sequence (mammary tumor and squamous cell carcinoma-associated (p80/85 src substrate) |
| 0.43 | PRO1073 | AA827878 | PRO1073 protein |
| 0.43 | KIAA0924 | AL036450 | KIAA0924 protein |
| 0.43 |  | BE327722 | Clone IMAGE:5314623, mRNA |
| 0.43 | KIAA0256 | AK022100 | KIAA0256 gene product |
| 0.43 | MYCN | BC002712 | v-myc myelocytomatosis viral related oncogene, neuroblastoma derived (avian) |
| 0.42 | C6orf63 | AI871589 | chromosome 6 open reading frame 63 |
| 0.42 | WASL | BE504979 | Wiskott-Aldrich syndrome-like |
| 0.41 | DIO2 | AI038059 | deiodinase, iodothyronine, type II |
| 0.41 | B3GAT2 | AA835648 | beta-1,3-glucuronyltransferase 2 (glucuronosyltransferase S) |
| 0.40 |  | R45950 | yg31b12.s1 Soares infant brain 1NIB Homo sapiens cDNA clone IMAGE:33958 3', mRNA sequence. |
| 0.40 |  | AI733194 | Transcribed sequences |
| 0.40 |  | AW452971 | Transcribed sequence with moderate similarity to protein pir:I60307 (E. coli) I60307 beta-galactosidase, alpha peptide - Escherichia coli |
| 0.38 | TOB1 | BF240286 | transducer of ERBB2, 1 |
| 0.37 |  | AI680875 | Sarcoma antigen NY-SAR-79 mRNA, partial cds |
| 0.37 | HBS1L | AI801875 | HBS1-like (S. cerevisiae) |
| 0.36 |  | BC026287 | Clone IMAGE:4770655, mRNA |
| 0.34 | EBAF | NM_003240 | endometrial bleeding associated factor (left-right determination, factor A; transforming growth factor beta superfamily) |
| 0.34 |  | AW274468 | Transcribed sequences |
| 0.33 | RBMS3 | AI821998 | RNA binding motif, single stranded interacting protein |
| 0.33 | HNRPD | AW005670 | heterogeneous nuclear ribonucleoprotein D (AU-rich element RNA binding protein 1, 37kDa) |
| 0.32 |  | BE465103 | Transcribed sequences |
| 0.30 | PRO1073 | NM_014086 |  |
| 0.29 |  | AI278445 | Transcribed sequence with weak similarity to protein sp:P39189 (H.sapiens) ALU2_HUMAN Alu subfamily SB sequence contamination warning entry |
| 0.27 | MSI2 | BE672557 | musashi homolog 2 (Drosophila) |
| 0.25 | C5orf15 | AI198794 | HTGN29 protein |
| 0.16 |  | BC020911 | Clone IMAGE:4732650, mRNA |
